# Enrichment of the tumour immune microenvironment in patients with desmoplastic colorectal liver metastasis

**DOI:** 10.1038/s41416-020-0881-z

**Published:** 2020-05-18

**Authors:** Diederik J. Höppener, Pieter M. H. Nierop, Joost Hof, Kostandinos Sideras, Guoying Zhou, Lydia Visser, Annette S. H. Gouw, Koert P. de Jong, Dave Sprengers, Jaap Kwekkeboom, Peter B. Vermeulen, Dirk J. Grünhagen, Cornelis Verhoef

**Affiliations:** 1 Department of Surgical Oncology and Gastrointestinal Surgery, Erasmus MC Cancer Institute, Rotterdam, The Netherlands; 2 Department of Hepato-Pancreato-Biliary Surgery and Liver Transplantation, University Medical Centre Groningen, University of Groningen, Groningen, The Netherlands; 3 Department of Gastroenterology and Hepatology, Erasmus Medical Centre, Rotterdam, The Netherlands; 4 Department of Pathology and Medical Biology, University Medical Centre Groningen, University of Groningen, Groningen, The Netherlands; 5 Translational Cancer Research Unit (GZA Hospitals and University of Antwerp), Antwerp, Belgium

**Keywords:** Cancer microenvironment, Prognostic markers, Colorectal cancer

## Abstract

**Background:**

Patients with resected colorectal liver metastasis (CRLM) who display only the desmoplastic histopathological growth pattern (dHGP) exhibit superior survival compared to patients with any non-desmoplastic growth (non-dHGP). The aim of this study was to compare the tumour microenvironment between dHGP and non-dHGP.

**Methods:**

The tumour microenvironment was investigated in three cohorts of chemo-naive patients surgically treated for CRLM. In cohort A semi-quantitative immunohistochemistry was performed, in cohort B intratumoural and peritumoural T cells were counted using immunohistochemistry and digital image analysis, and in cohort C the relative proportions of individual T cell subsets were determined by flow cytometry.

**Results:**

One hundred and seventeen, 34, and 79 patients were included in cohorts A, B, and C, with dHGP being observed in 27%, 29%, and 15% of patients, respectively. Cohorts A and B independently demonstrated peritumoural and intratumoural enrichment of cytotoxic CD8+ T cells in dHGP, as well as a higher CD8+/CD4+ ratio (cohort A). Flow cytometric analysis of fresh tumour tissues in cohort C confirmed these results; dHGP was associated with higher CD8+ and lower CD4+ T cell subsets, resulting in a higher CD8+/CD4+ ratio.

**Conclusion:**

The tumour microenvironment of patients with dHGP is characterised by an increased and distinctly cytotoxic immune infiltrate, providing a potential explanation for their superior survival.

## Background

Colorectal cancer (CRC) represents one of the most common solid malignancies.^1^ Metastatic spread occurs in roughly half of all patients during the course of the disease, with colorectal liver metastasis (CRLM) presenting as the most frequent distant metastasis.^2–5^ Depending on the hepatic tumour load and vessel involvement, local therapies, often in conjunction with systemic therapy, selectively allow for curatively intended treatment strategies, even in the case of limited extrahepatic metastatic disease.^6^ Herein surgical resection is often considered the mainstay treatment modality.^7^ Reported 5-year overall survival (OS) rates after curatively intended surgical treatment for CRLM generally range from 40% to 60%.^8,9^

Prognostication and prediction of treatment effect after surgical treatment of CRLM has changed little over time and is still based mainly on clinicopathological factors, most notably the nodal status of the primary tumour, the number and size of hepatic metastases, and RAS mutational status.^10–16^ Only in mismatch repair-deficient tumours, which account for roughly 3% of patients with CRLM^17^, has a clear therapeutic indication been demonstrated for immune checkpoint inhibitors.^18,19^ This clearly emphasises the need for additional, clinically relevant biomarkers. To this end, recent efforts have focussed on the quantification and classification of immune cells present within the tumour microenvironment (TME) of CRC and/or CRLM.^20–26^ Results have been promising, with favourable prognosis demonstrated in patients with increased and activated (i.e. cytotoxic) immune infiltrates in the TME.^20–26^

**Fig. 1:**
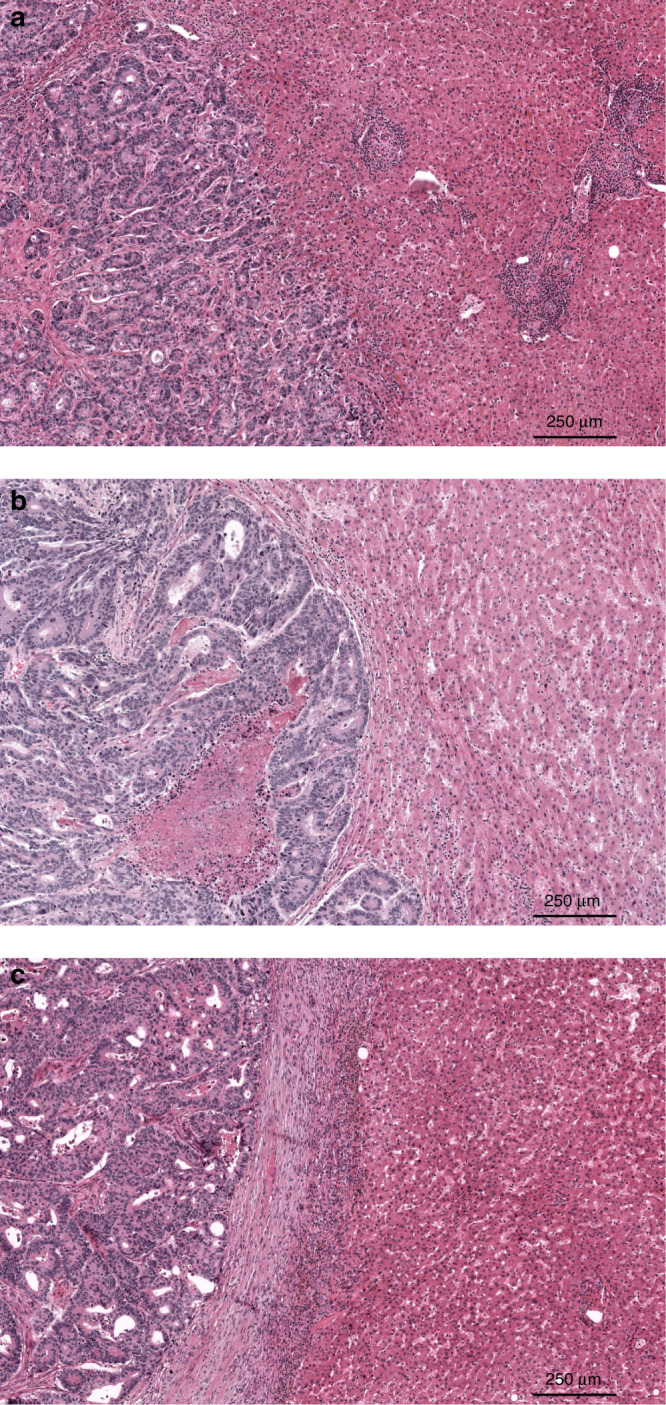
The histopathological growth patterns (HGP) of colorectal liver metastasis on haematoxylin and eosin-stained tissue sections. **a** The replacement HGP (rHGP), **b** the pushing HGP (pHGP), and **c** the desmoplastic HGP (dHGP).

Another emerging biomarker encompassing the TME is the histopathological growth pattern (HGP) of CRLM. The HGPs describe the morphology and interaction between tumour and liver cells at the tumour–liver interface.^27^ Histomorphologically, three phenotypes are distinguished: the replacement type, where the tumour cells “replace” liver cells while the sinusoidal architecture is maintained at the tumour–liver interface (Fig. 1a); the rare pushing type, where the tumour cells “push” against the liver cell plates (Fig. 1b); and the desmoplastic type (dHGP), where a band of desmoplastic stroma separates the tumour from the liver parenchyma (Fig. 1c). Apart from these apparent differences upon histomorphological examination, the desmoplastic and pushing types have angiogenic ways of vascularisation, while the replacement type relies on vessel co-option.^27–31^ For all that, clinical relevance seems determined by two classes: either patients where tumours are fully enclosed by a desmoplastic rim (i.e. 100% dHGP) or patients where any non-desmoplastic (i.e. <100% dHGP; non-dHGP) pattern is observed, as multiple HGPs can appear in conjunction.^32^ Especially in chemo-naive subjects (i.e. not treated with systemic chemotherapy within 6 months prior to resection of CRLM), patients with dHGP exhibit superior survival compared to their non-dHGP counterparts, with reported 5-year OS rates of nearly 80% in dHGP and as low as 40% in (any) non-dHGP.^32^

Upon histomorphological examination, dHGP is often characterised by a distinct immune infiltrate surrounding the desmoplastic stroma (Fig. 1c), although this has never been quantified, classified, or been compared to non-dHGP counterparts in light of the discovery of the any non-dHGP on/off phenomenon.^32^ The aim of the current study was therefore to quantify, classify, and compare the TME of CRLM between patients with dHGP and non-dHGP. Given there is evidence to suggest that systemic therapy not only affects the immune infiltrate in the TME^26,33^ but also influences the proportional distribution and possibly the prognostic value of the HGPs^32^, our study focussed on chemo-naive subjects only.

## Methods

Investigation of the TME of CRLM was performed in three cohorts, each analysed using distinct methods. Scoring of the HGPs of CRLM was performed similarly across all cohorts and according to international consensus guidelines.^27^ The current study was approved by the medical ethics committee of the Erasmus Medical Centre (MEC-2018-1743).

### Scoring of the HGP of CRLM

In each of the three cohorts, all available haematoxylin and eosin (H&E)-stained slides of formalin-fixed paraffin-embedded tissue blocks of resected CRLM specimens were retrieved from the archives of the respective pathology departments. Scoring of the HGP was performed retrospectively using either light microscopy or digitalised slide images. All available and eligible (digitalised) tissue sections were reviewed by simultaneous assessment of at least two trained observers. For all tissue sections subjected to review, the relative percentage of each distinct HGP (i.e. pushing, desmoplastic, and replacement type) was determined at the tumour–liver interface. Given recent findings by Galjart et al.^32^, patients were classified as dHGP if only the desmoplastic type was observed in all the reviewed sections (i.e. 100% dHGP, Fig. 1c) and as non-dHGP if any pushing and/or replacement type was observed in any of the reviewed sections (i.e. <100% dHGP, Fig. 1a, b).

### Cohort A: semi-quantitative immunohistochemistry (IHC)

In the first cohort, analysis of the TME of CRLM was performed using semi-quantitative IHC in patients who underwent partial hepatectomy with curative intent at either the Erasmus MC Cancer Institute, Rotterdam, the Netherlands or the University Medical Centre Groningen (UMCG), Groningen, the Netherlands. Patients eligible for inclusion were those with complete metastasectomy (defined as resection margin >0 mm), who did not receive any preoperative and/or postoperative chemotherapy in addition to partial hepatectomy, a Clinical Risk Score^10^ (CRS) of ≤3, no extrahepatic disease at the time of surgery, and no known medical history of secondary malignancy. Data on this cohort, together with RNA sequencing experiments performed in the UMCG cohort only, has previously been submitted for publication (submitted manuscript). IHC staining was performed on 4-µm-thick tissue sections cut from formalin-fixed paraffin-embedded samples of resected CRLM (Supplementary Fig. 1). For each formalin-fixed paraffin-embedded sample, a control slide was stained for H&E to confirm the presence of tumourous and adjacent liver tissue. IHC staining for CD4 (SP35), CD8 (SP57), CD45 (RP2/18), CD79A (SP18), and Kappa/Lambda (double polyclonal staining) was done using the Ventana automated staining system (Roche, Basel, Switzerland). Manual staining was performed with the primary antibodies FoxP3 (236A/E7, 1/100 dilution) and SLAMF7 (HPA055945, 1/200 dilution). Positive and negative controls were implemented. All IHC-stained tissue sections were assessed by two trained observers. Expression was graded semi-quantitatively ranging from 1 to 3 and was determined for peritumoural and intratumoural regions separately. Peritumoural was defined as the expression observed at the tumour–liver interface, and intratumoural was defined as expression observed in the stroma surrounding the tumour cells or immunopositive intraepithelial lymphocytes. After consensus was reached by both observers, expression of each marker was classified into “low” and “high” using the cut-off value resulting in the most even distribution (1 vs ≥2 or ≤2 vs 3). In addition, the CD8-to-CD4 ratio was calculated by dividing their respective semi-quantitative scores (i.e. grades 1–3) for the peritumoural and intratumoural regions separately. A “high” CD8 to CD4 ratio was defined as a ratio greater or equal to the median.

### Cohort B: quantitative IHC by digital image analysis

Analysis of the TME in the second cohort consisted of quantitative IHC using digital image analysis. Patients were eligible if they underwent partial hepatectomy with curative intent at the Erasmus MC Cancer Institute, Rotterdam, the Netherlands and if they did not receive any systemic chemotherapy treatment in the 6 months prior to resection. This cohort represents a subset of a larger cohort that has previously been published.^25^ IHC staining for CD8 (SP57) and FoxP3 (236A/E7, 1/100 dilution) was performed on 4-µm-thick tissue sections using the Ventana Benchmark Ultra automated staining system (Roche, Basel, Switzerland). Stained tissue sections were digitalised at 40× using the NanoZoomer 2.0HT system (Hamamatsu Photonic, Shizuoka, Japan). Peritumoural and intratumoural cell densities of CD8+ and FoxP3+ were measured in cells/mm^2^ using the Visiopharm Integrator System (version 4.2.2.0, Visiopharm, Hoersholm, Denmark). Peritumoural cell densities were determined in four high-power fields (0.54 mm in diameter) at the tumour–liver interface (Supplementary Fig. 2). The intratumoural cell densities were determined in several (4–6) large circular areas containing viable tumourous tissue (Supplementary Fig. 2). In addition, the CD8+/FoxP3+ ratio was determined for peritumoural and intratumoural densities separately.

### Cohort C: flow cytometry

In the third cohort, the TME was analysed using flow cytometry. Patients eligible for inclusion were those who underwent partial hepatectomy at the Erasmus MC Cancer Institute, Rotterdam, the Netherlands and if they did not receive any systemic chemotherapy treatment in the 6 months prior to resection. Data on (part of) this cohort has previously been published.^22,23,25^ The relative proportions of CD4+ T cells, CD4+FoxP3− T helper cells, CD4+FoxP3+ T regulatory cells, and CD8+ cytotoxic T cells within live CD3+ T cells were determined by flow cytometry in mononuclear cells (MNCs) isolated from fresh tumour tissue, tumour-free liver (obtained as distant as possibly from the tumour; minimum 1 cm distance), and in peripheral blood mononuclear cells (PMBCs) isolated from peripheral blood collected prior to surgery. Ficoll density gradient centrifugation was used for PBMC isolation. Single-cell suspensions from tumour and tumour-free liver were obtained by tissue digestion. Fresh tissue was cut into small pieces and digested for 30 min at 37 °C with interrupted gentle swirling either in PRMI 1640 medium (Lonza, Breda, the Netherlands) with 0.5 mg/ml collagenase IV (Sigma-Aldrich, St. Louis, MO, USA) and 0.1 mg/ml DNase I (Roche, Basel, Switzerland) or in Hanks’ Balanced Salt solution with Ca^2+^ and Mg^2+^ (Sigma, Zwijndrecht, the Netherlands) with 0.125 mg/ml collagenase IV and 0.2 mg/ml DNase I.^22,23^ Filtration of cell suspensions was done through 100-µm pore cell strainers (BD Biosciences, Franklin Lakes, NJ, USA). Ficoll density gradient centrifugation was used to obtain MNCs. Viability was determined by trypan blue exclusion. Cells were surface-labelled with fluorochrome-conjugated antibodies against CD45 (optional), CD3, CD4, and CD8. Intracellular FoxP3 was stained using FoxP3-specific antibody (clone 236A/E7; eBioscience, San Diego, CA, USA) after fixation and permeabilisation using the FoxP3 staining buffer set of eBioscience (San Diego, CA, USA). Subsequent flow cytometric analysis was performed using a FACS Canto II flow cytometer (BD Biosciences, Franklin Lakes, NJ, USA) and FlowJo software (version 10.0, BD, Franklin Lakes, NJ, USA) as described previously.^22,23^ Viable (aqua LIVE/DEAD fluorescent dye-negative) leukocytes were gated in single cells using either CD45 or FSC/SSC. Live T cells were defined based on CD3 expression. Within live CD3+ T cells, the relative proportions of CD8+ and CD4+ T cell subsets were determined. Within the CD4+ T cells, the T regulatory subset was defined as CD4+FoxP3+ while the T helper subset was defined as CD4+FoxP3−. In addition to these subsets, the ratio between CD8+/CD4+ T cells and the ratio between CD4+FoxP3−/CD4+FoxP3+ T cells were calculated. A representative example of the flow cytometry gating strategy is provided in Supplementary Fig. 3. The study was approved by the medical ethics committee of the Erasmus Medical Centre (MEC-2012-331) and signed informed consent was obtained from all patients prior to tissue and blood donation.

### Survival

The OS, defined as the time in months from resection of CRLM till death, was compared between patients with dHGP and non-dHGP in all the three cohorts combined.^32^ Overall survival was estimated by Kaplan–Meier method and reported as 5-year OS rate with corresponding 95% confidence interval (CI). Survival curves were compared using log-rank test.

### Statistical analysis

The TME was compared in each cohort between patients who exhibited only dHGP (i.e. 100% dHGP) and patients in whom any non-dHGP was observed (i.e. <100% dHGP). Categorical data were compared using chi-squared test and non-parametric continuous data using Kruskal–Wallis test. In addition, linear regression was performed to study possible relations between the observed percentage of dHGP at the tumour–liver interface and the TME. Herein the total proportion of dHGP observed at the tumour–liver interface represented the independent variable, and continuous data observed in the TME the dependent outcome variable. In order to test whether the HGP and the TME were independent of clinical risk, the CRS was determined.^10^ Patients were classified as either low (CRS 0–2) or high (CRS 3–5) risk. Independency of the HGP with CRS was tested for all cohorts combined and for each cohort separately. Independency with CRS was also tested for CD8, CD4, and FoxP3 within each cohort. Categorical data are reported as frequency and/or percentage and plotted using bar charts with binomial 95% CI. When plotted, binomial 95% CI for proportions were calculated using the Clopper–Pearson method. Non-parametric continuous data are reported as median with corresponding 25th (Q1) and 75th (Q3) percentile (i.e. interquartile range (IQR)) and plotted using box plots. Outliers in box plots were defined according to the 1.5 rule (i.e. outside [Q1 − 1.5 × IQR; Q3 + 1.5 × IQR]). Statistical significance was defined as an *α* < 0.05. All statistical analyses were performed using R version 3.5.3 (http://www.r-project.org).

## Results

Data on 198 individual patients were collected, 160 of whom received treatment at the Erasmus MC Cancer Institute, and the remaining 38 at the University Medical Centre Groningen. Of the 160 patients of the Erasmus MC, 1 was included in all three cohorts, 2 were included in both cohorts A and C, and 28 were included in both cohorts B and C. Upon histopathological examination, dHGP was observed in 46 patients (23%). The CRS was available for 191 patients (98%) and was independent of the HGP (*p* = 0.089, Supplementary Table 1). Clinicopathological patient characteristics stratified by cohort are reported in Table 1.

**Table 1:** Baseline characteristics stratified by cohort.

		Cohort A	Cohort B	Cohort C
		Semi-quantitative IHC	Quantitative IHC	Flow cytometry
		*n* = 117 (%)	*n* = 34 (%)	*n* = 79 (%)
Centre	Erasmus MC	79 (68)	34 (100)	79 (100)
	UMCG	38 (32)	0 (0)	0 (0)
Age at resection of CRLM (median [IQR])		67.0 [61.0, 73.0]	64.5 [57.2, 72.0]	67.0 [59.0, 75.0]
Gender	Female	50 (43)	11 (32)	29 (37)
	Male	67 (57)	23 (68)	50 (63)
Primary tumour location	Left sided	48 (41)	17 (50)	40 (51)
	Right sided	48 (41)	11 (32)	25 (32)
	Rectal	21 (18)	5 (15)	11 (14)
	Missing	0 (0)	1 (3)	3 (4)
Adjuvant CTx following CRC resection	No	85 (73)	31 (91)	63 (80)
	Yes	30 (26)	3 (9)	15 (19)
	Missing	2 (2)	0 (0)	1 (1)
Nodal status of primary CRC	N0	59 (50)	18 (53)	39 (49)
	N+	58 (50)	14 (41)	39 (49)
	Missing	0 (0)	2 (6)	1 (1)
Disease-free interval in months^a^ (median [IQR])		15.0 [4.0, 25.0]	7.0 [0.0, 17.5]	8.0 [0.0, 18.5]
Preoperative CEA in µg/L (median [IQR])		16.1 [4.6, 50.5]	6.0 [3.9, 17.0]	13.0 [5.6, 29.1]
Number of CRLM (median [IQR])		1.0 [1.0, 2.0]	2.0 [1.0, 2.0]	1.0 [1.0, 2.0]
Diameter of largest CRLM in cm (median [IQR])		3.4 [2.5, 4.5]	2.4 [1.5, 3.5]	3.0 [2.0, 3.8]
Clinical Risk Score	Low risk (0–2)	101 (86)	22 (65)	57 (72)
	High risk (3–5)	16 (14)	8 (24)	17 (22)
	Missing	0 (0)	4 (12)	5 (6)
Histopathological growth pattern	dHGP	32 (27)	10 (29)	12 (15)
	non-dHGP	85 (73)	24 (71)	67 (85)

*CEA* carcinoembryonic antigen, *CRC* colorectal cancer, *CRLM* colorectal liver metastasis, *dHGP* desmoplastic-type histopathological growth pattern, *IHC* immunohistochemistry, *IQR* interquartile range, *non-dHGP* non-desmoplastic-type histopathological growth pattern, *UMCG* University Medical Centre Groningen.

^a^Between resection of primary CRC and detection of CRLM.

### Cohort A: semi-quantitative IHC

**Fig. 2:**
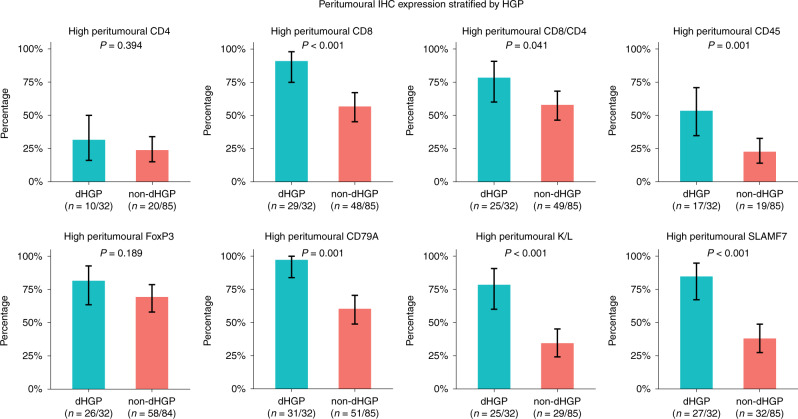
Results of semi-quantitative immunohistochemistry (IHC) in cohort A: bar charts representing the proportion of patients with high peritumoural expression of individual markers stratified by histopathological growth pattern (HGP). The black lines represent the binomial 95% confidence interval (Clopper–Pearson). K/L Kappa/Lambda.

**Fig. 3:**
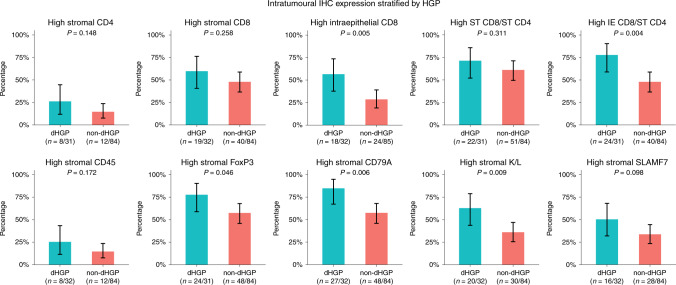
Results of semi-quantitative immunohistochemistry (IHC) in cohort A: bar charts representing the proportion of patients with high intratumoural expression of individual markers stratified by histopathological growth pattern (HGP). The black lines represent the binomial 95% confidence interval (Clopper–Pearson). ST stromal, IE intraepithelial, K/L Kappa/Lambda.

A total of 117 patients were included in the first cohort, 79 of whom underwent resection of CRLM at the Erasmus MC Cancer Institute between March 2000 and February 2015 and 38 of whom underwent resection of CRLM at the University Medical Centre Groningen between January 1994 and June 2013. Clinicopathological patient characteristics are reported in Table 1. Thirty-two patients exhibited dHGP (27%) and 85 non-dHGP (73%). The results of peritumoural and intratumoural IHC expression stratified by HGP are reported in Figs. 2 and 3, respectively. All intratumoural expression was scored based on stromal expression, with the exception of CD8, which was determined on both stromal and intraepithelial lymphocyte expression. The cut-off to determine “high” expression was grade 3 for CD4 and CD45 and grade ≥2 for all other markers. The TME of dHGP patients more often displayed high peritumoural CD8, CD45, CD79A, Kappa/Lambda, and SLAMF7 expression, all *p* ≤ 0.001 (Fig. 2). Similarly, high intratumoural CD8 (intraepithelial), CD79A, FoxP3, and Kappa/Lambda were more frequently observed in the TME of patients with dHGP, all *p* < 0.05 (Fig. 3). Concerning the CD8-to-CD4 ratio, patients with dHGP more often displayed a high peritumoural CD8/CD4 (*p* = 0.041, Fig. 2), as well as a high intraepithelial CD8 to stromal CD4 (*p* = 0.004, Fig. 3). No difference was found in the stromal CD8 to stromal CD4 ratio (*p* = 0.311, Fig. 3). Peritumoural and intratumoural CD8, CD4, and FoxP3 expression were all independent of CRS (all *p* > 0.10, Supplementary Table 1).

### Cohort B: quantitative IHC by digital image analysis

**Fig. 4:**
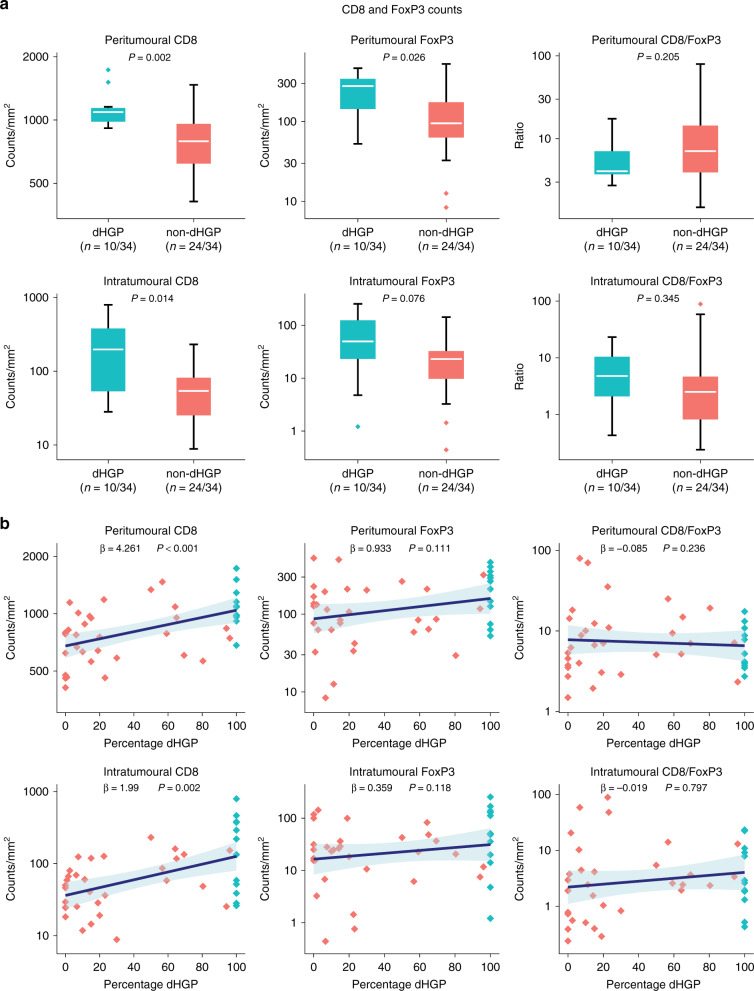
Results of quantitative immunohistochemistry in cohort B. **a** Box and whisker plots of intratumoural and peritumoural counts/mm^2^ stratified by histopathological growth pattern (HGP) and displayed on a logarithmic scale. The white line represents the median, the box represents the interquartile range (IQR), and the whisker represents the range. Outliers are defined according to the 1.5 rule (i.e. outside [Q1 − 1.5 × IQR; Q3 + 1.5 × IQR]). **b** Linear regression models of intratumoural and peritumoural counts/mm^2^ (*y*-axis, logarithmic scale) and the percentage of the desmoplastic-type histopathological growth pattern (dHGP) scored at the tumour–liver interface (*x*-axis). The blue line represents the regression coefficient; the light blue ribbon represents the corresponding 95% confidence interval. Measurements of individual patients are displayed using dots. Red dots represent patients with non-dHGP (i.e. <100% dHGP), and blue dots represent patients with dHGP (i.e. 100% dHGP).

Quantitative IHC by digital image analysis was performed in 34 patients who underwent partial hepatectomy at the Erasmus MC Cancer Institute between October 2009 and October 2011. Clinicopathological patient characteristics are reported in Table 1. Out of 34, dHGP was observed in 10 (29%) and non-dHGP in 24 (71%) patients. Figure 4a reports the peritumoural and intratumoural CD8 and FoxP3 counts stratified by HGP using box plots. The TME of dHGP patients was associated with significantly higher peritumoural and intratumoural CD8 (*p* = 0.002 and *p* = 0.014, respectively) and peritumoural FoxP3 counts (*p* = 0.026). No significant difference was observed concerning intratumoural FoxP3 counts or the peritumoural and intratumoural CD8/FoxP3 ratios. Figure 4b displays the linear regression models investigating the peritumoural and intratumoural CD8 and FoxP3 counts and the total percentage of dHGP at the tumour–liver interface. The percentage of dHGP at the tumour–liver interface proved a significant positive predictor for both peritumoural (*β* = 4.261, *p* < 0.001) and intratumoural (*β* = 1.99, *p* = 0.002) CD8 counts. No such associations were found for peritumoural and intratumoural FoxP3 counts or the peritumoural and intratumoural CD8/FoxP3 ratios (all *p* > 0.10, Fig. 4b). Peritumoural and intratumoural CD8 and FoxP3 counts were all independent of CRS (all *p* > 0.15, Supplementary Table 1).

### Cohort C: flow cytometry

**Fig. 5:**
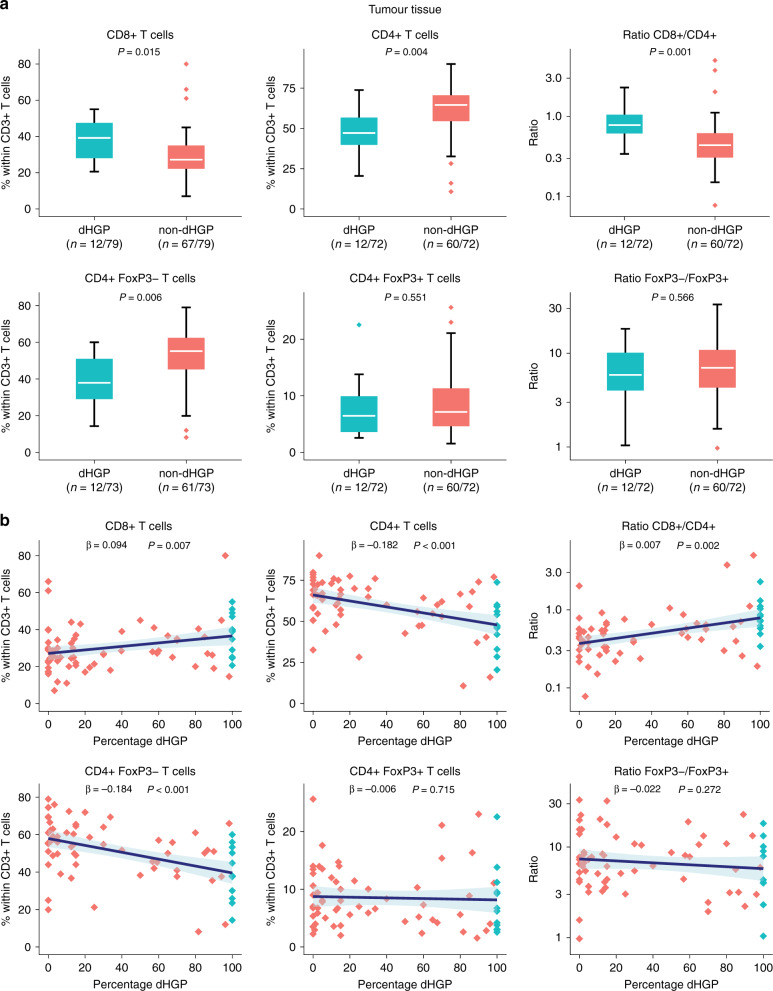
Results of flow cytometry of fresh tumour samples in cohort C. **a** Box and whisker plots of the relative proportion of individual T cell subsets stratified by histopathological growth pattern (HGP). Ratios are displayed on a logarithmic scale. The white line represents the median, the box represents the interquartile range (IQR), and the whisker represents the range. Outliers are defined according to the 1.5 rule (i.e. outside [Q1 − 1.5 × IQR; Q3 + 1.5 × IQR]). **b** Linear regression models of the relative proportion of individual T cell subsets (*y*-axis) and the percentage of the desmoplastic-type histopathological growth pattern (dHGP) scored at the tumour–liver interface (*x*-axis). Ratios are displayed on a logarithmic scale. The blue line represents the regression coefficient; the light blue ribbon represents the corresponding 95% confidence interval. Measurements of individual patients are displayed using dots. Red dots represent patients with non-dHGP (i.e. <100% dHGP), and blue dots represent patients with dHGP (i.e. 100% dHGP).

Viable MNCs were successfully isolated from tumour tissue of 79 patients who underwent partial hepatectomy at the Erasmus MC Cancer Institute between October 2009 and August of 2018. Viable MNCs from tumour-free liver tissue were successfully isolated in 73 and viable PBMCs from peripheral blood samples in 55 of the 79 patients. Clinicopathological patient characteristics are reported in Table 1. Twelve (15%) patients were found to have dHGP; non-dHGP was seen in 67 patients (85%). Figure 5a reports the relative proportions of T cell subsets within CD3+ T cells isolated from tumour tissue stratified by HGP using box plots. The relative proportion of CD8+ T cells was significantly higher in patients with dHGP (*p* = 0.015), while the relative proportion of CD4+ T cells was significantly higher in patients with non-dHGP (*p* = 0.004). Congruently, the CD8/CD4 ratio was significantly higher in dHGP patients (*p* = 0.001). This difference in CD4+ T cells was due to a higher relative CD4+FoxP3− T helper subset in non-dHGP only (*p* = 0.006), as no difference was observed for the CD4+FoxP3+ regulatory T cell subset (*p* = 0.551). Concerning the CD4+FoxP3−/CD4+FoxP3+ ratio, no difference was observed (*p* = 0.566). Similar results were seen in the linear regression models investigating T cell subsets in tumour tissue and the percentage of dHGP at the tumour–liver interface, reported in Fig. 5b. A positive linear association was found for the percentage of dHGP and the CD8+ T cell subset (*β* = 0.094, *p* = 0.007), while a negative linear association was seen for the CD4+ T cell subset (*β* = −0.182, *p* < 0.001). Correspondingly, the percentage of dHGP was positively associated with the CD8+/CD4+ ratio (*β* = 0.007, *p* = 0.002). Within the CD4+ subsets, the percentage of dHGP was only negatively associated with the CD4+FoxP3− subset (*β* = −0.184, *p* < 0.001), as no association was found between dHGP and the CD4+FoxP3+ subset (*p* = 0.715). No linear association was found for the CD4+FoxP3−/CD4+FoxP3+ ratio (*p* = 0.272). The relative proportions of CD8+, CD4+, CD4+FoxP3−, and CD4+FoxP3+ T cells in fresh tumour tissues were all independent of CRS (all *p* > 0.30, Supplementary Table 1).

The relative proportions of T cell subsets within CD3+ T cells in tumour-free liver tissues and peripheral blood stratified by HGP, as well as the linear regression models investigating T cell subsets in tumour-free liver or peripheral blood and the percentage of dHGP at the tumour–liver interface, are reported in Supplementary Figs. 4a, 5a, 4b, and 5b, respectively. When stratifying for HGP, no differences existed in the relative proportions of T cell subsets in either tumour-free liver tissues (all *p* > 0.30, Supplementary Fig. 4a) or peripheral blood samples (all *p* > 0.50, Supplementary Fig. 5a). Interestingly, the percentage of dHGP at the tumour–liver interface was negatively associated with the relative proportion of CD4+ T cells in tumour-free liver samples (*β* = −0.103, *p* = 0.021, Supplementary Fig. 4b), and positively associated with the CD8+/CD4+ ratio (*β* = 0.022, *p* = 0.026, Supplementary Fig. 4b). Again, this association was due to the CD4+FoxP3− T helper subset (*β* = −0.099, *p* = 0.018, Supplementary Fig. 4b), as no association existed for the CD4+FoxP3+ regulatory T cell subset (*β* = −0.003, *p* = 0.371, Supplementary Fig. 4b). No association was found for the CD8+ subset or the CD4+FoxP3−/CD4+FoxP3+ ratio (both *p* > 0.10, Supplementary Fig. 4b). Concerning possible associations between T cell subsets in peripheral blood samples and the percentage of dHGP at the tumour–liver interface, no relationships were found (all *p* > 0.20, Supplementary Fig. 5b).

### Survival

**Fig. 6:**
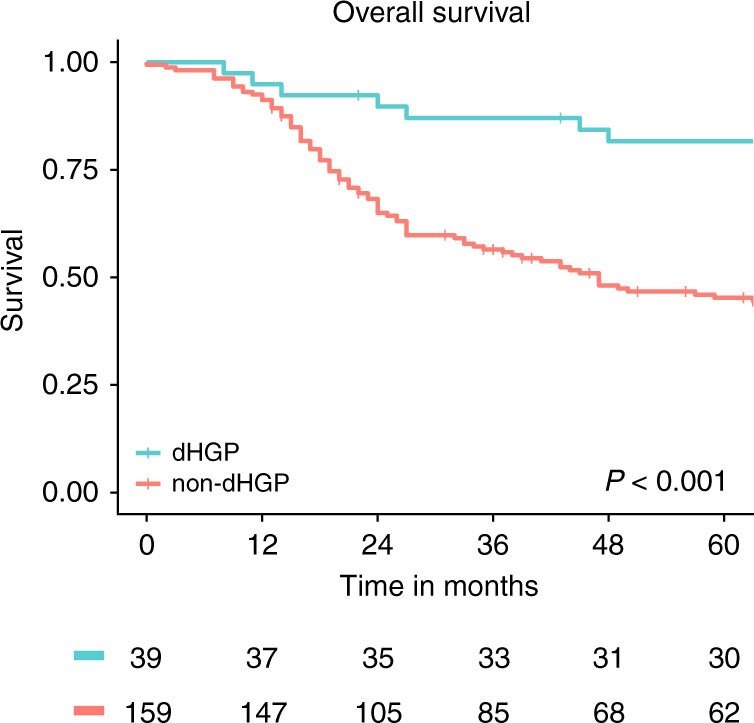
Kaplan–Meier overall survival estimates stratified by histopathological growth pattern (HGP) in all the three cohorts combined. The numbers at risk are displayed in the table below. *p* Value represents overall log-rank test.

Survival data was available for all 198 patients. The Kaplan–Meier estimates for OS stratified by HGP are reported in Fig. 6. The 5-year OS (95% CI) rate for patients with dHGP was 82% (70–95%) compared to 45% (38–54%) for non-dHGP (overall log rank: *p* < 0.001).

## Discussion

The current study aimed to quantify, classify, and compare the TME of patients with dHGP and non-dHGP. Three distinct analytic methods were applied in 3 cohorts of chemo-naive patients undergoing resection of CRLM (with 1 patient included in all 3 cohorts, 2 included in both cohorts A and C, and 28 in both cohorts B and C). In order to correctly interpret the results, it is important to recognise the difference in outcome measures of each analytical method.

In the first cohort (A), semi-quantitative IHC scoring was applied. High peritumoural expression of CD8, CD45, CD79A, Kappa/Lambda, and SLAMF7 and intratumoural CD8 (intraepithelial), CD79A, Kappa/Lambda, and FoxP3 were significantly more often seen in patients with dHGP. In addition, dHGP was associated with a high peritumoural CD8/CD4 ratio, as well as a high intraepithelial CD8 to stromal CD4 ratio. These differences suggest a general increased immune infiltrate in dHGP, both in the peritumoural and the intratumoural TME. In cohort A, intratumoural CD8 was determined for both stromal and intraepithelial expression. Interestingly, high intraepithelial CD8 expression was more often seen in dHGP patients (*p* = 0.005), whereas no significant difference was found for stromal CD8 expression (*p* = 0.258). Intraepithelial CD8+ lymphocytes have been linked to favourable prognosis in CRC and are associated with antitumour immunity.^34^ Furthermore, it has been postulated that intraepithelial CD8+ lymphocytes play an important role in the suppression of micrometastasis and hence are associated with a decrease in distant metastasis.^34^ The higher expression of intraepithelial CD8+ lymphocytes in patients with dHGP therefore corroborates the recent findings that patients with non-dHGP are at higher risk for extrahepatic and multi-organ recurrences following first surgical treatment of CRLM.^35^

This general increased immune infiltrate in patients with dHGP seen in cohort A is supported by cohort B, where CD8 and FoxP3 expression levels were quantified both peritumourally and intratumourally using digital image analysis. Median counts/mm^2^ of peritumoural CD8 and FoxP3 and intratumoural CD8 were significantly higher in dHGP patients. Previously published results describing a cohort that consisted of the same patients plus patients treated with preoperative chemotherapy suggested that the CD8/FoxP3 ratio was prognostic for survival after resection of CRLM.^25^ Given the superior survival observed in chemo-naive dHGP patients^32^, one would expect dHGP to be associated with a high CD8/FoxP3 ratio. Contrastingly, no relationship between the HGP and the CD8/FoxP3 ratio was found.

Assuming that a general increased immune infiltrate is present in dHGP, interpretation of individual markers from cohorts A and B is somewhat difficult due to the non-relative nature of their outcome. Relative increases in the TME of non-dHGP patients could exist but—given the absence of normalisation methods for individual IHC markers—are potentially missed when analysing just expression grades or absolute cell counts. Furthermore, IHC analysis does not always allow for adequate discrimination between individual cell populations, for instance, concerning CD4, which is expressed on both CD4+FoxP3− T helper cells as CD4+FoxP3+ T regulatory cells. In the third cohort, analysis was performed using flow cytometry, which incorporates a relative outcome measure (i.e. proportion within live CD3+ T cells) and allows for discrimination of T helper and T regulatory cell populations.

Flow cytometry in fresh tumour tissues showed a relative increase in CD8+ T cells within infiltrated CD3+ T cells in the TME of patients with dHGP. Contrastingly, the TME of non-dHGP patients was associated with a relative increase in CD4+ T cells within infiltrating CD3+ T cells. These results are in line with cohort A, considering the semi-quantitative nature of the outcome measure and that CD4 was the *only* marker in which no significant difference in either intratumoural or peritumoural expression existed between dHGP and non-dHGP. The relative increase in CD8 and relative decrease in CD4 in the TME of dHGP patients was consistent with the CD8/CD4 ratio, which was significantly higher in patients with dHGP in both cohorts A and C. The relative increase of CD4+ T cells in the TME of non-dHGP patients appeared only due to an increased CD4+FoxP3− T helper subset, as no difference was found for the CD4+FoxP3+ T regulatory subset. This is especially interesting considering the previously demonstrated immunosuppressive effect of CD4+FoxP3+ T regulatory cells on antitumour immunity.^22,36^ The absence of a difference in relative numbers of T regulatory cells within CD3+ T cells in the TME between dHGP and non-dHGP patients suggests that the detrimental prognosis observed in non-dHGP patients may *not* be mediated by T regulatory cells (or at least T regulatory cell numbers, since functionality was not studied). The flow cytometric data show that the observed increases in absolute numbers of FoxP3+ cells observed in cohorts A and B are probably due to increased absolute number of T cells in the TME of dHGP patients and not to selective enrichment of the regulatory T cell subset within infiltrating CD3+ T cells.

While the T cell immune infiltrate was investigated in all three cohorts, B and plasma cells were only investigated in the first. Herein CD79A, a double polyclonal Kappa/Lambda, and SLAMF7 staining were used to identify B lineage and plasma cells. As stated before, interpretation of these individual markers should be done with caution due to absent normalisation. Nevertheless, some of the most striking differences in both the peritumoural and intratumoural TME in cohort A were observed in the expression of these B/plasma cell markers. For instance, high peritumoural CD79A was observed in all but 1 (97%) of the patients with dHGP vs 60% in non-dHGP and high intratumoural CD79A in >80% vs <60%. Similarly, large differences were seen for intratumoural and peritumoural Kappa/Lambda and peritumoural SLAMF7. While T cells in CRC (metastases) have been studied extensively, less is known about the prognostic impact of B and plasma cells. A recent review identified five studies investigating the prognostic impact of CD20+ B cell tumour infiltration within CRC (metastases).^37^ Three of these studies demonstrated a positive,^38–40^ one demonstrated a negative,^41^ and one failed to demonstrate any prognostic effect of tumour-infiltrating CD20+ B cells.^42^ The majority of studies in other cancer types also report positive prognostic effects of tumour-infiltrating B cells.^37^ It is thought that B cell production of stimulatory cytokines can enhance the T cell antitumour response.^37^ In addition, the production of tumour antigen-specific antibodies by plasma cells could trigger antibody-dependent cellular cytotoxicity and enhance antigen presentation to T cells through Fc receptors on dendritic cells.^37^ It has, however, also been suggested that B and plasma cell infiltration is the result of interferon-γ production and might therefore be more a reflection of the T cell antitumour response rather than a mediating factor.^43^ Although only demonstrated in a single cohort by a single method, these results suggest that the TME of dHGP could also be characterised by B cell and plasma cell enrichment. Further research should aim at validating these findings and to determine underlying mechanisms.

Assimilation of all three cohorts demonstrates an increased absolute and relative infiltration of CD8+ cytotoxic T cells in the TME of patients with dHGP. This provides a potential explanation to the superior survival previously observed in patients with dHGP^32^ and also within the current study. All the more because the HGP and the immune infiltrate at the TME were found to be independent of clinical risk. Not only has increased infiltration of CD8+ cytotoxic T cells been linked to prognosis in primary CRC^44^ and metastatic CRC patients^24,40,45^ but Katz and colleagues have also specifically correlated increased CD8+ T cell infiltration to prolonged survival following resection of CRLM.^21^ Brunner et al. also found that high CD8+ infiltration was linked to favourable prognosis in patients with CRLM.^46^ Moreover, Brunner et al. specifically correlated fibrotic capsule formation (which likely represents the 100% dHGP population of our study) with high CD8+, CD45+, and CD4+ infiltration on IHC, suggesting a general increased immune infiltrate in those patients.^46^ This is similar to our results from cohorts A and B of the current study, where a general increased immune infiltrate in dHGP was found. This general increased immune infiltrate further adds to the possible explanation for the superior survival observed in dHGP, since Brunner et al. specifically reported that an increased immune infiltrate, especially in combination with fibrotic capsule formation, was strongly related to favourable prognosis.^46^ Likewise, Katz et al. reported a general increased infiltration of CD3 T cells to be prognostic following surgical treatment of CRLM.^21^ More recently, the internationally validated immunoscore for stage I–III CRC proposed by Galon et al.^20,47^, derived from intratumoural and peritumoural densities of CD3+ and CD8+ T cells, was also found to be positively correlated with favourable prognosis in patients with CRLM.^26^

The question then arises whether the immune response seen in the TME drives the HGP phenotype (i.e. HGPs are host-determined) or that intrinsic tumour characteristics determine the HGP, in turn driving the immune phenotype (i.e. HGPs are tumour-driven). Linear regression analysis in cohorts B and C demonstrated a positive linear relationship between the percentage of dHGP scored at the tumour–liver interface and CD8+ T cells, as well as the CD8/CD4 ratio. In addition, a negative linear association for CD4+ T cells existed, which was explained by a negative linear association in the CD4+FoxP3− T helper cell subset only. These linear relationships indicate a level of interactivity between the immune infiltrate and the HGP phenotype. Considering that flow cytometry of distant tumour-free liver samples demonstrated similar linear relationships between CD4+ T cells, CD4+FoxP3− T helper cells, the CD8/CD4 ratio, and the percentage of dHGP scored at the tumour–liver interface, HGPs could, at least in part, be host-determined. Linear regression analysis of peripheral blood samples and the percentage of dHGP showed no such linear correlations, suggesting that the HGP phenotype may be more influenced by the local immunologic environment of the liver than by systemic immunity.

The strength of our study is that three cohorts were independently studied using distinct analytic methods. However, some limitations have to be noted. First, data on intrinsic tumour characteristics such as mismatch repair status and RAS/RAF mutational status were unavailable in all three cohorts. It would have been especially valuable to include mismatch repair status, since it is currently the only indication for checkpoint inhibitors within metastatic CRC. Consequently, mismatch repair status is thought to be a main driving force of the immune infiltrate.^18,19^ Mismatch repair deficiency is, however, only present in 3% of the patients with CRLM.^17^ As such, mismatch repair deficiency alone could never account for the entire dHGP phenotype, which is present in roughly 20% of chemo-naive CRLM patients^32^, suggesting (at least partial) independency. Second, although HGP evaluation was performed according to international consensus guidelines^27^, assessment was done by several observers and both light microscopy and digitalised slide images were used. This is likely of little relevance though, as interobserver reliability for HGP assessment, even for trained observers with limited histopathological experience, was found to be excellent.^48^ Furthermore, within- and between-metastasis concordance of HGPs is especially high in chemo-naive patients.^48^ Third, the semi-quantitative IHC assessment in cohort A only incorporated grading of antibody expression and not antibody intensity compared to positive control. Previous studies have incorporated methods for scoring both antibody expression and antibody intensity.^49^ Such methods would have likely added discriminatory power in cohort A and is something that should be considered for similar future investigations. Finally, flow cytometry could only be performed in samples from which sufficient viable MNCs for flow cytometric analysis could successfully be isolated. Thus patients with a desert immune phenotype are likely not included in the analyses. No data were available on the frequency of unsuccessful isolation of viable MNCs from tumour, tumour-free liver, or peripheral blood samples. In addition, CD45 was not always included in the flow cytometry panel due to limited channels. It would be interesting to compare CD3+ T cells (and its subsets) based on the CD45+ population.

In conclusion, the current study demonstrates that the TME of chemo-naive patients with a purely angiogenic desmoplastic growth pattern is characterised by a general increased and distinctly cytotoxic immune infiltrate compared to patients with any observed non-desmoplastic growth. These findings provide a potential explanation for the superior survival observed in chemo-naive patients with purely desmoplastic colorectal liver metastases.

## Supplementary information

Supplementary Table 1

Supplementary Figure 1

Supplementary Figure 2

Supplementary Figure 3

Supplementary Figure 4

Supplementary Figure 5

## Data Availability

The data sets generated and/or analysed during the current study are not publicly available but are available upon request and at the discretion of the corresponding author.
