# Supplementary material for: Enrichment of the tumour immune microenvironment in patients with desmoplastic colorectal liver metastasis
Source: Br J Cancer. 2020 May 18;123(2):196–206. doi: 10.1038/s41416-020-0881-z (PMC7374625; doi:10.1038/s41416-020-0881-z)
Supplement: Supplementary file 1 — Supplementary Table 1 [file 41416_2020_881_MOESM1_ESM.pdf]

Supplementary table 1. Comparison of the HGP and the TME by clinical risk score.

		Clinical risk score Low risk (CRS 0-2)	Clinical risk score High risk (CRS 3-5)	
All patients		n = 158 (%)	n = 33 (%)	p-value
Histopathological growth pattern	dHGP	41 (26)	4 (12)	0.089
	non-dHGP	117 (74)	29 (88)	
Cohort A: semi-quantitative IHC		n = 101 (%)	n = 16 (%)	p-value
Histopathological growth pattern	dHGP	30 (30)	2 (12)	0.151
	non-dHGP	71 (70)	14 (88)	
CD8 - high peritumoural		67 (66)	10 (62)	0.764
CD8 - high stromal		48 (48)	11 (69)	0.123
CD8 - high intratumoural		36 (36)	6 (38)	0.886
CD4 - high peritumoural		28 (28)	2 (12)	0.195
CD4 - high stromal		17 (17)	3 (19)	0.877
FoxP3 - high peritumoural		73 (73)	11 (69)	0.724
FoxP3 - high stromal		63 (64)	9 (56)	0.571
Cohort B: quantitative IHC		n = 22 (%)	n = 8 (%)	p-value
Histopathological growth pattern	dHGP	7 (32)	2 (25)	0.719
	non-dHGP	15 (68)	6 (75)	
CD8 - intratumoural counts/mm^2 - *(median [IQR])*		50.9 [26.1, 77.4]	76.4 [55.8, 168.0]	0.159
CD8 - peritumoural counts/mm^2 - *(median [IQR])*		918.2 [694.6, 1093.3]	892.0 [702.7, 1257.4]	0.851
FoxP3 - intratumoural counts/mm^2 - *(median [IQR])*		24.1 [11.8, 46.8]	32.0 [5.4, 103.0]	0.888
FoxP3 - peritumoural counts/mm^2 - *(median [IQR])*		126.5 [75.1, 254.4]	150.2 [52.5, 366.2]	0.851
Cohort C: flow cytometry (fresh tumour tissues)		n = 57 (%)	n = 17 (%)	p-value
Histopathological growth pattern	dHGP	10 (18)	2 (12)	0.570
	non-dHGP	47 (82)	15 (88)	
% CD8+ T-cells* - *(median [IQR])*		27.2 [22.3, 37.0]	28.5 [24.5, 34.4]	0.928
% CD4+ T-cells* - *(median [IQR])*		62.0 [47.2, 69.2]	60.5 [56.7, 69.9]	0.363
% CD4+ FoxP3- T-cells* - *(median [IQR])*		51.3 [38.5, 61.0]	54.7 [50.6, 59.9]	0.317
% CD4+ FoxP3+ T-cells* - *(median [IQR])*		6.7 [4.0, 10.0]	8.8 [5.6, 11.4]	0.371

* Expressed as percentage of CD3+ T-cells gate

Abbreviations in alphabetical order: CRS: *clinical risk score;* dHGP: *desmoplastic type histopathological growth pattern;* HGP: *histopathological growth pattern;* IHC: *immunohistochemistry;* IQR: *interquartile range;* non-dHGP: *non-desmoplastic type histopathological growth pattern;* TME: *tumour microenvironment* .
