# Supplementary material for: Enrichment of the tumour immune microenvironment in patients with desmoplastic colorectal liver metastasis
Source: Br J Cancer. 2020 May 18;123(2):196–206. doi: 10.1038/s41416-020-0881-z (PMC7374625; doi:10.1038/s41416-020-0881-z)
Supplement: Supplementary file 2 — Supplementary Figure 1 [file 41416_2020_881_MOESM2_ESM.pdf]

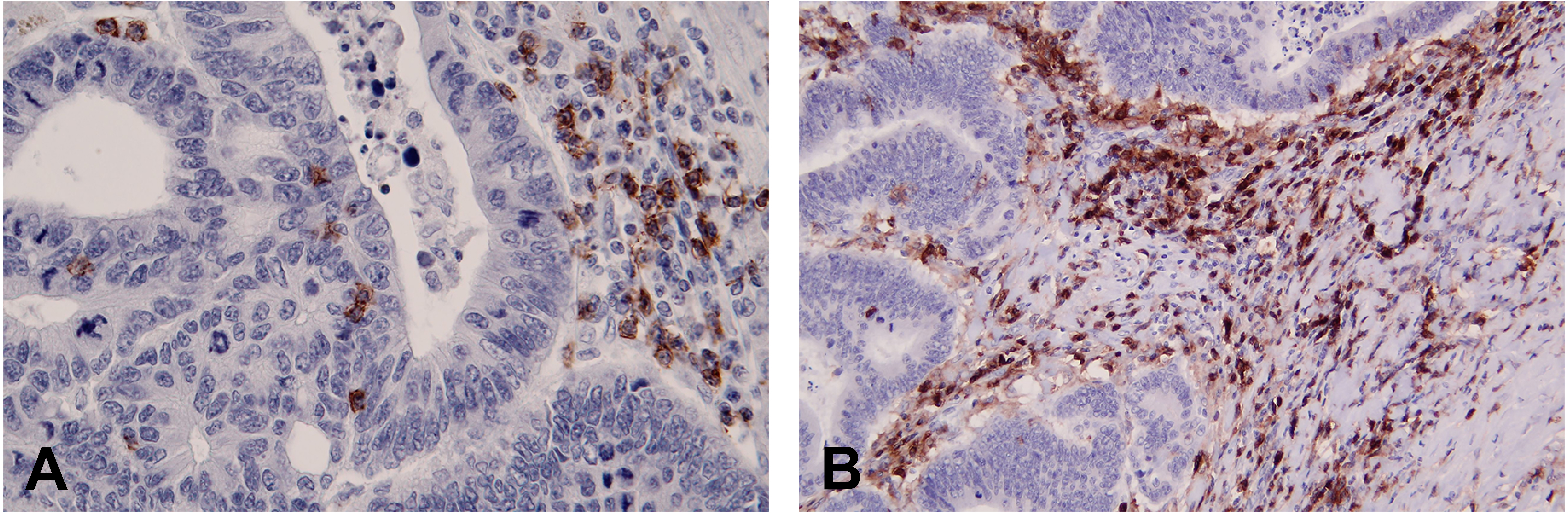

Supplementary figure 1: examples of immunohistochemistry (IHC) staining used in cohort A. **(A)** CD8 IHC staining. **(B)** CD4 IHC staining.
