# Supplementary material for: Enrichment of the tumour immune microenvironment in patients with desmoplastic colorectal liver metastasis
Source: Br J Cancer. 2020 May 18;123(2):196–206. doi: 10.1038/s41416-020-0881-z (PMC7374625; doi:10.1038/s41416-020-0881-z)
Supplement: Supplementary file 3 — Supplementary Figure 2 [file 41416_2020_881_MOESM3_ESM.pdf]

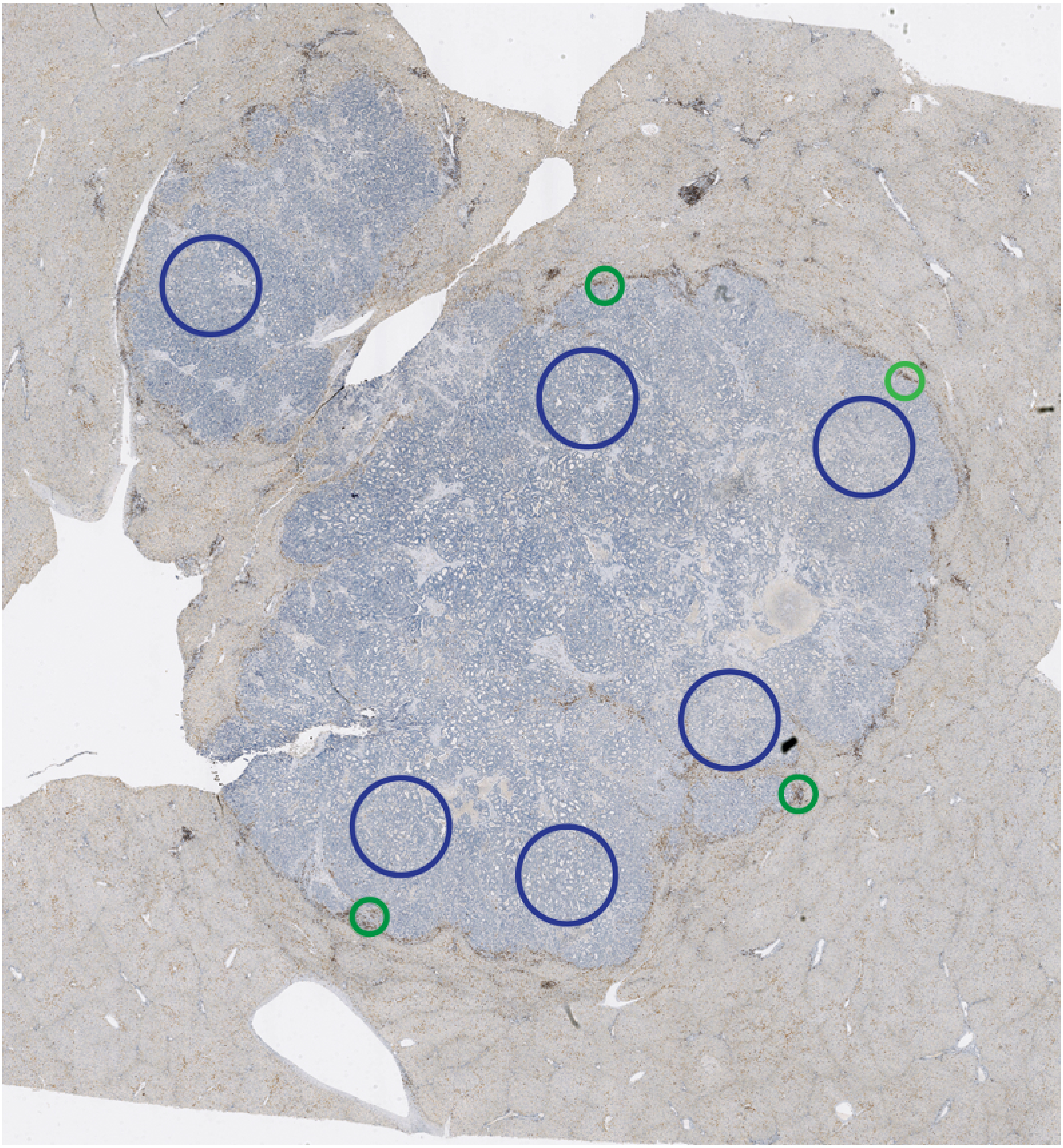

Supplementary figure 2: quantitative immunohistochemistry using digital image analysis in cohort B. Intratumoural cell densities were determined in several (4-6) large circular areas containing viable tumorous tissue (blue circles). The peritumoural cell densities were determined in four high-power fields (0.54mm in diameter) at the tumour-liver interface (green circles).
