# Supplementary material for: Enrichment of the tumour immune microenvironment in patients with desmoplastic colorectal liver metastasis
Source: Br J Cancer. 2020 May 18;123(2):196–206. doi: 10.1038/s41416-020-0881-z (PMC7374625; doi:10.1038/s41416-020-0881-z)
Supplement: Supplementary file 4 — Supplementary Figure 3 [file 41416_2020_881_MOESM4_ESM.pdf]

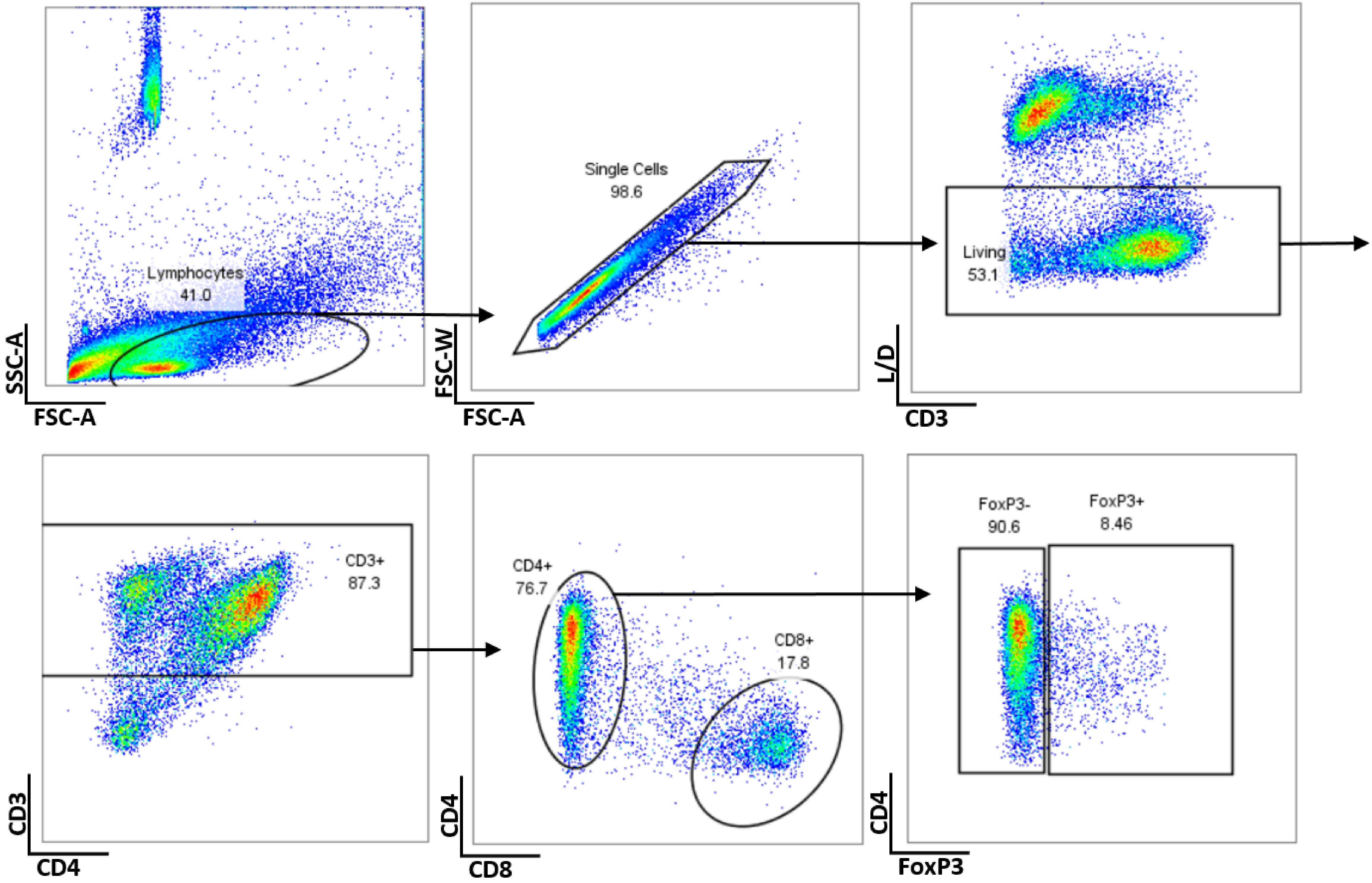

Supplementary figure 3: representative example of the flow cytometry gating strategy (top-left to bottom-right) in cohort C. Flow cytometric analysis was performed using a FACS Canto II flow cytometer and FlowJo software. Viable (aqua LIVE/DEAD [L/D] fluorescent dye-negative) leukocytes were gated in single cells using FSC and SSC. Live T-cells were defined based on CD3 expression. Within live CD3+ T-cells, the relative proportions of CD8+ and CD4+ T-cell subsets were determined. Within the CD4+ T-cells, the T-regulatory subset was defined as CD4+FoxP3+ while the T-helper subset was defined as CD4+FoxP3-. SSC-A: side scatter area; FSC-A: forward scatter area; FSC-W: forward scatter width.
