# Supplementary material for: Enrichment of the tumour immune microenvironment in patients with desmoplastic colorectal liver metastasis
Source: Br J Cancer. 2020 May 18;123(2):196–206. doi: 10.1038/s41416-020-0881-z (PMC7374625; doi:10.1038/s41416-020-0881-z)
Supplement: Supplementary file 5 — Supplementary Figure 4 [file 41416_2020_881_MOESM5_ESM.pdf]

Tumour−free liver tissue

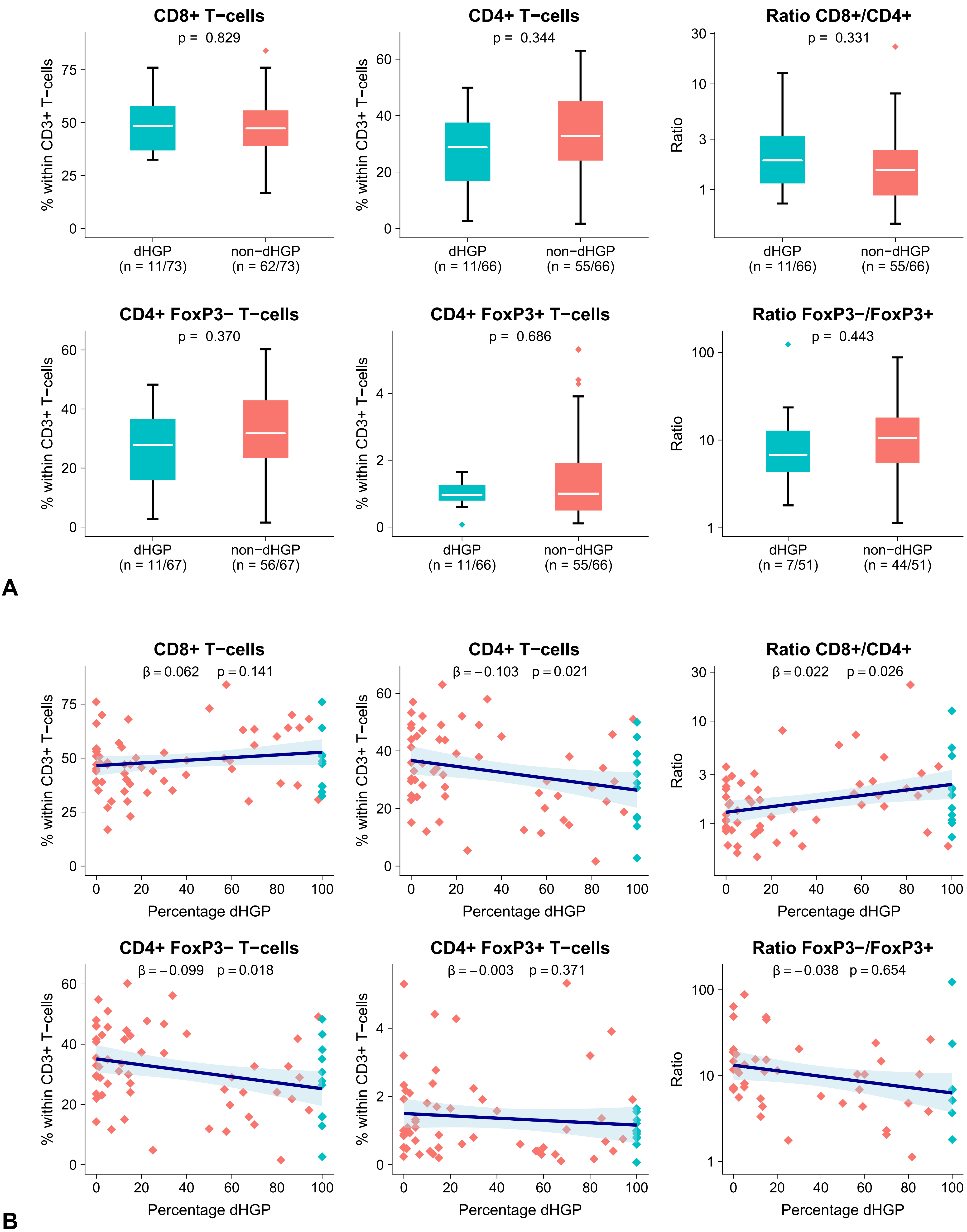

Supplementary figure 4: results of flow cytometry of fresh tumour-free liver samples in cohort C. **(A)** Box and whiskerplots of the relative proportion of individual T-cell subsets stratified by histopathological growth pattern (HGP). Ratio's are displayed on a logarithmic scale. The white line represents the median, the box represents the inter-quartile rage (IQR), and the whiskers represents the range. Outliers are defined according to the 1.5 rule (i.e. outside [Q1-1.5*IQR; Q3+1.5*IQR]). **(B)** Linear regression models of the relative proportion of individual T-cell subsets (y-axis) and the percentage of the desmoplastic type histopathological growth pattern (dHGP) scored at the tumour-liver interface (x-axis). Ratio's are displayed on a logarithmic scale. The blue line represents the regression coefficient, the lightblue ribbon represents the corresponding 95% confidence interval. Measurements of individual patients are displayed using dots. Red dots represent patients with non-dHGP (i.e. < 100% dHGP) and blue dots represent patients with dHGP (i.e. 100% dHGP).
